# Advancing modified barium swallow pre-sorting with deep learning: a new paradigm for the first step analysis in X-ray swallowing study

**DOI:** 10.1007/s11548-025-03505-y

**Published:** 2025-10-04

**Authors:** Shitong Mao, Mohamed A. Naser, Sheila Buoy, Kristy K. Brock, Katherine A. Hutcheson

**Affiliations:** 1 Department of Head and Neck Surgery, University of Texas MD Anderson Cancer Center, Houston, TX 77030 USA; 2 Department of Radiation Oncology, Division of Radiation Oncology, University of Texas MD Anderson Cancer Center, Houston, TX 77030 USA; 3 Department of Imaging Physics, Division of Diagnostic Imaging, University of Texas MD Anderson Cancer Center, Houston, TX 77030 USA

**Keywords:** Modified barium swallow, Deep learning, Convolutional neural networks, Swallowing disorders, Dysphagia, Videofluoroscopy

## Abstract

**Purpose:**

Modified barium swallow (MBS) exams are pivotal for assessing swallowing function and include diagnostic video segments imaged in various planes, such as anteroposterior (AP or coronal plane) and lateral (or mid-sagittal plane), alongside non-diagnostic ‘scout’ image segments used for anatomic reference and image set-up that do not include bolus swallows. These variations in imaging files necessitate manual sorting and labeling, complicating the pre-analysis workflow.

**Methods:**

Our study introduces a deep learning approach to automate the categorization of swallow videos in MBS exams, distinguishing between the different types of diagnostic videos and identifying non-diagnostic scout videos to streamline the MBS review workflow. Our algorithms were developed on a dataset that included 3,740 video segments with a total of 986,808 frames from 285 MBS exams in 216 patients (average age 60 ± 9).

**Results:**

Our model achieved an accuracy of 99.68% at the frame level and 100% at the video level in differentiating AP from lateral planes. For distinguishing scout from bolus swallowing videos, the model reached an accuracy of 90.26% at the frame level and 93.86% at the video level. Incorporating a multi-task learning approach notably enhanced the video-level accuracy to 96.35% for scout/bolus video differentiation.

**Conclusion:**

Our analysis highlighted the importance of leveraging inter-frame connectivity for improving the model performance. These findings significantly boost MBS exam processing efficiency, minimizing manual sorting efforts and allowing raters to allocate greater focus to clinical interpretation and patient care.

## Introduction

**Fig. 1:**
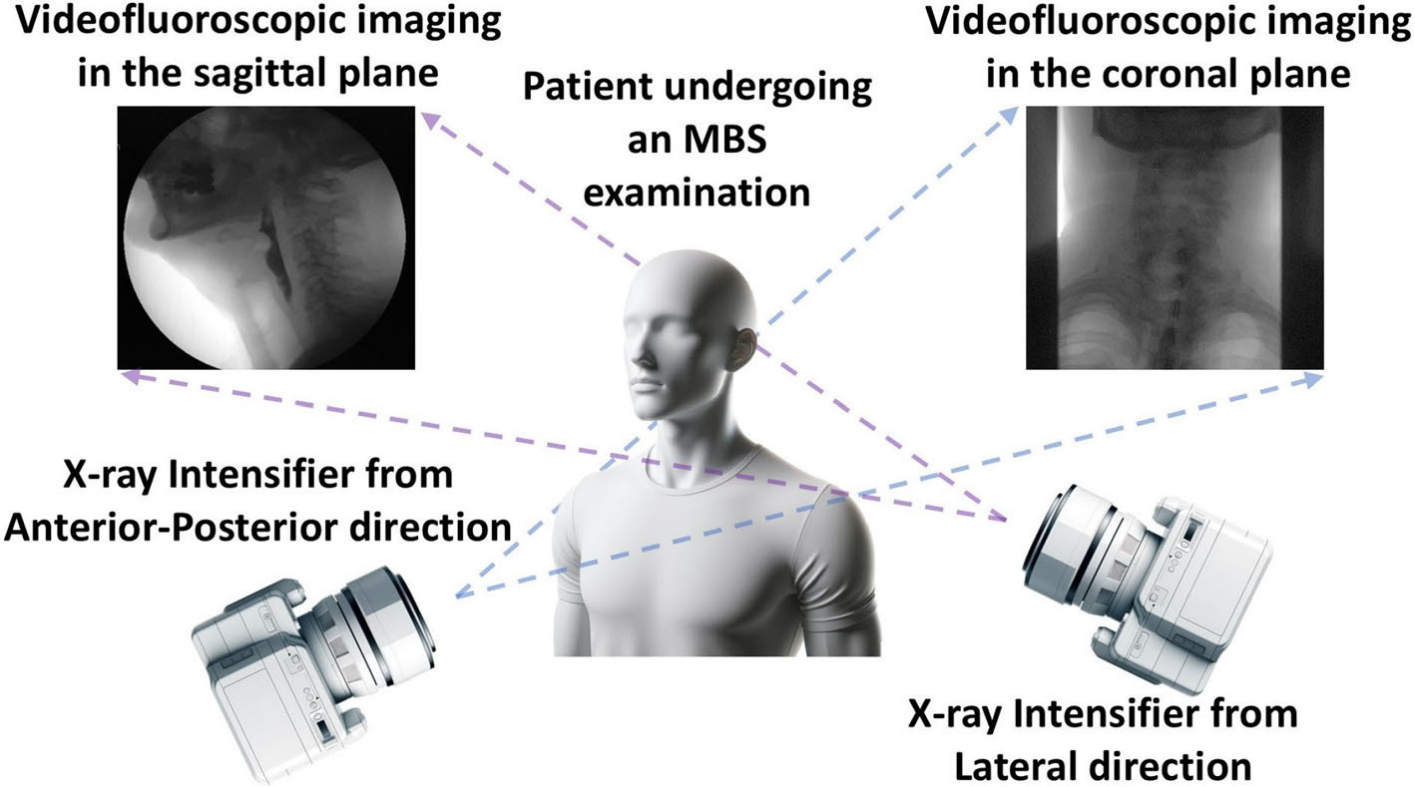
Configuration of MBS examination that includes AP and lateral orientations corresponding to the coronal and sagittal planes, respectively

The modified barium swallow (MBS) study is a dynamic X-ray imaging technique employing videofluoroscopy. MBS is a workhorse in clinical assessment of dysphagia, a condition of swallowing difficulty. MBS provides invaluable insights into the biomechanics of swallowing by visualizing key structures in the digestive tract during a swallow. The exam is also used to assess the safety (keeping food/liquids out of the airway) and efficiency (clearing food/liquids fully through the throat) of swallow function. In a typical MBS exam, a series of swallowing trials is conducted using barium as the contrast agent. Each trial is meticulously recorded, often saved as individual video segments or clips (corresponding to the on/off timing of fluoroscopy), with the trials executed in both lateral and anteroposterior (AP) planes, as shown in Fig. 1. This approach facilitates comprehensive visualization in the sagittal plane for lateral views and the coronal plane for AP views, respectively. The majority of swallowing trials are captured in the lateral orientation, providing a detailed view of the swallowing mechanism, including airway protection and bolus clearance. They are the basis for evidence-based clinical measurements, like the Penetration-Aspiration Scale (PAS) [1], Dynamic Imaging Grade of Swallowing Toxicity (DIGEST) [2, 3], and Modified Barium Swallow Impairment Profile [4, 5]. Despite the predominance of lateral views, AP orientation trials are equally important, offering unique insights into the symmetry of the swallowing process and the width of pharyngeal and esophageal lumen.

Also, within an MBS study, ‘scout’ videos (also called initial, preliminary, baseline, or setup views [6]) are often captured at the outset of an MBS without barium. These trials provide anatomic variations such as swelling, anterior cervical osteophytes, or implants that might cause artifacts or affect swallowing and bolus flow [7, 8]. Another purpose is to fine-tune the field of view and imaging settings prior to barium swallows and confirm that key landmarks (e.g., hyoid bone, cervical vertebrae, hypopharynx) are visible, ensuring the entire swallowing mechanism is properly recorded in subsequent trials [9]. Additional ‘scout’ may be taken during the MBS study if repositioning or equipment adjustments are needed, or if the anatomy must be re-evaluated without contrast. In practice, these non-diagnostic scout videos are often recorded and mixed with diagnostic swallowing clips due to the sequential nature of MBS exams.

In MBS studies, video clips must be recorded for detailed post-exam analysis. Slow motion and frame-by-frame review are critical because many swallowing events happen within milliseconds [10]. However, sorting poses a major challenge: Non-diagnostic ‘scout’ videos must be manually excluded. While videos captured in the AP direction are still relevant and must be identified, many MBS-derived dysphagia measurements (e.g., PAS and DIGEST) use only lateral clips, so clinicians need to accurately categorize orientations. Manual sorting is manageable for a single study, but grows more complex with larger datasets. Filtering out non-diagnostic videos and identifying AP clips from potentially hundreds or thousands of recordings, to isolate those suitable for clinical measurements, is both time-consuming and labor-intensive.

Recent advances in computer vision and deep learning have significantly propelled automated MBS exam analyses for clinical outcomes. Studies have investigated deep learning algorithms for dynamic anatomical landmarks detection in videofluoroscopy, including hyoid bone detection and tracking [11, 12], food bolus segmentation [13, 14], and cervical vertebrae localization [15], as well as classifying the PAS and assessing dysphagia [16, 17]. These efforts represented considerable progress toward establishing a comprehensive, time-efficient, clinically dependable, and economically viable automated system for swallowing imaging studies. Nonetheless, these algorithms inherently relied on meticulously curated datasets, explicitly excluding ‘scout’ and AP direction videos. For full MBS analysis, the impact and effectiveness of applying existing algorithms to AP video frames remain uncertain. Similarly, automated bolus segmentation without an actual bolus also remains untested. Presently, manual video pre-sorting is the only approach for data pre-processing, contradicting the goal of a fully automated diagnostic process. The reliance on manual video sorting represents a fundamental impediment toward seamless integration of advanced computational techniques in the practical implementation, necessitating innovative solutions to bridge this gap in dysphagia-focused imaging advancements.

To tackle these challenges, we propose a deep learning-based methodology to automate the pre-analysis sorting of swallowing videos in MBS studies. Our method focuses on two critical tasks: classifying video clips by orientation (AP or lateral) and distinguishing ‘scout’ from bolus swallowing clips. This study elaborates on the development and validation of our methods, assessing their performance at both frame and video levels. We also investigate the advantages of a multi-task learning strategy to enhance the model’s precision. The results demonstrate the model’s effectiveness in accurately categorizing various types of swallowing videos, marking a significant stride in the integration of AI-driven methodologies in the realm of computational deglutition [18].

## Methods

### Data collection

In this study, a dataset was collected from 216 patients (average age 60.24 ± 9.02 years, including 192 males) at the University of Texas, MD Anderson Cancer Center from 2016 to 2022. The analysis of these MBS videos was approved by the Institutional Review Board (IRB) of the University of Texas MD Anderson Cancer Center (IRB approval number PA19-0261, May 10, 2019). Each patient underwent a standard MBS exam with continuous fluoroscopy recorded at 30 FPS during approximately 10 barium bolus trials in lateral orientation and 2 bolus trials in AP orientation as a routine clinical procedure.

The MBS videos were manually annotated for two classification tasks, each reviewed by at least two independent raters in a video-by-video analysis approach. The first task classified video direction (AP, lateral, or unknown/uncertain). The second task identified ‘scout’ videos (scout, bolus swallowing, or unknown/uncertain). Exclusion criteria were applied as follows: (1) videos labeled as ‘unknown/uncertain’ by any rater, (2) rater disagreement, (3) mixed orientation frames within a single video, and (4) duplicate videos. The dataset contained a total of 285 MBS exams, yielding 3,740 video clips with a total of 986,808 frames. Details on data distribution are outlined in Table 1. The gender distribution was attributable to the clinical cohort characteristics, as the majority of patients in this study were diagnosed with head and neck cancer, which has been well investigated to exhibit a higher incidence in male individuals [19, 20].

**Table 1:** Dataset summary for deep learning algorithm development

Task	AP vs. Lateral	‘Scout’ vs. Bolus swallow
Patients number	208 (age 60.24 ± 8.99, 186 Males)	214 (age 60.14 ± 9.04, 190 Males)
Exam number	279	285
Video number	3,540 (AP: 490, lateral: 3,050)	3,689 (‘scout’: 220, bolus: 3,469)
Total frame number	939,799 (AP: 140,068, lateral: 799,731)	976,218 (‘scout’: 13,143, bolus: 963, 075)

### Deep learning model development

**Fig. 2:**
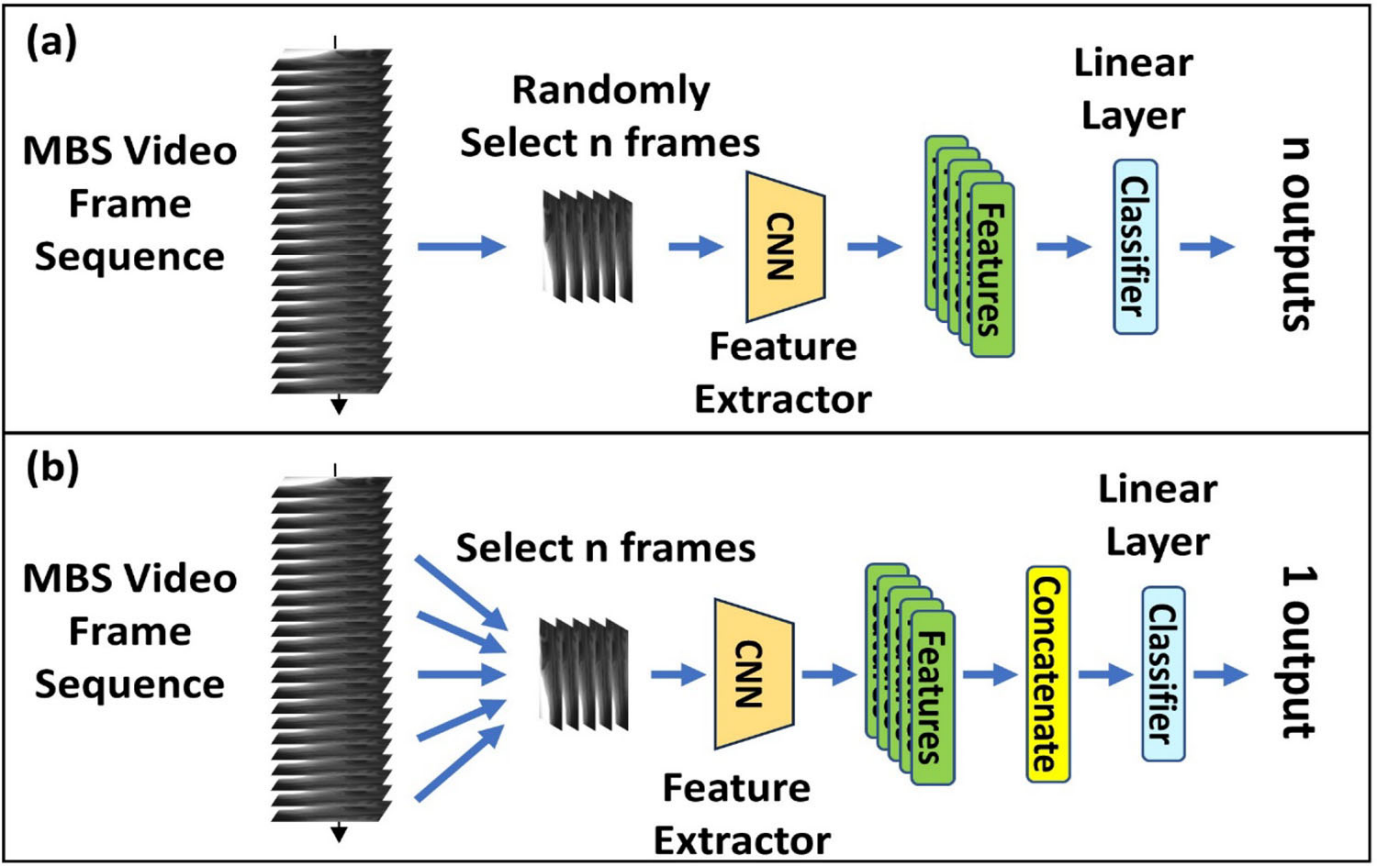
Schematic representation of the data processing strategies for deep learning model training. (**a**) Frame-wise analysis and (**b**) Video-wise analysis

In this study, we designed two data processing pipelines: frame-wise and video-wise, illustrated in Fig. 2. In the frame-wise approach, each video frame was treated as a separate entity with the video-level label applied to every frame. During training, we randomly selected a batch of videos and then randomly sampled a set number of frames (n, a hyperparameter) from each video. In the video-wise approach, we first sampled consecutive frames from a randomly chosen 75% segment of the video. We then divided these frames into (n − 1) equal intervals and picked n frames at these fixed points. A CNN (ResNet50) extracted features from these frames, which were flattened and concatenated into a single vector for video-level classification via a linear layer.

We evaluated both pipelines with varying numbers of frames (n) to examine how frame selection affected performance. ResNet50 was chosen for its robust feature extraction across diverse image recognition tasks [21]. For the following experiments, we varied the number of frames (n) to investigate the impact of frame count on model performance.

### Training and validation

In this study, we followed a cross-subject prediction approach for designating training, validation, and testing sets as outlined in [22]. Some patients had MBS videos annotated for both video orientation and ‘scout’ tasks, while others had annotations for only one. To avoid data leakage, we considered that patients included in the training set for one classification task must be excluded from the validation and testing sets. Consequently, the validation and testing sets comprised patients with MBS videos annotated for both tasks, ensuring dataset consistency and relevance. The data were split into training, validation, and testing sets at approximately 70%, 15%, and 15%, respectively. Table 2 provides detailed information on these datasets.

**Table 2:** The demographic distribution for the training, validation and testing sets

	Training set for video orientation task	Training set for ‘scout’/bolus swallowing videos	Validation set	Testing set
Patients	144	150	32	32
Age	59.80 ± 8.97	59.83 ± 9.04	62.59 ± 8.76	59.84 ± 8.94
Gender	M: 126, F: 18	M: 130, F: 20	M: 31, F: 1	M: 29, F: 3
Videos	2,489	2,638	530	521
Frames	668,756	705,175	148,797	122,246

Model optimization was conducted using the training dataset, with performance evaluated on the validation set at each epoch. We applied early stopping, ending training if validation accuracy did not improve after 50 epochs. The best-performing model on the validation set was then chosen for final testing. During testing, for frame-wise models, we first calculated performance at the frame level and then used majority voting for the final video classification (video-level results). For video-wise models, frames for testing were selected from the entire video without random segmentation. Given the methodology in Fig. 2, video-level performance metrics were exclusively calculated for the video-wise analysis approach. (Frame-wise analysis was unavailable.)

### Multi-task learning approach

We used multi-task learning to address video orientation classification and ‘scout’ vs. bolus swallowing tasks in this study, leveraging shared semantic information across frames. We hypothesize that insights from analyzing lateral orientation videos can improve ‘scout’ video identification, thereby enhancing model performance and efficiency.

**Fig. 3:**
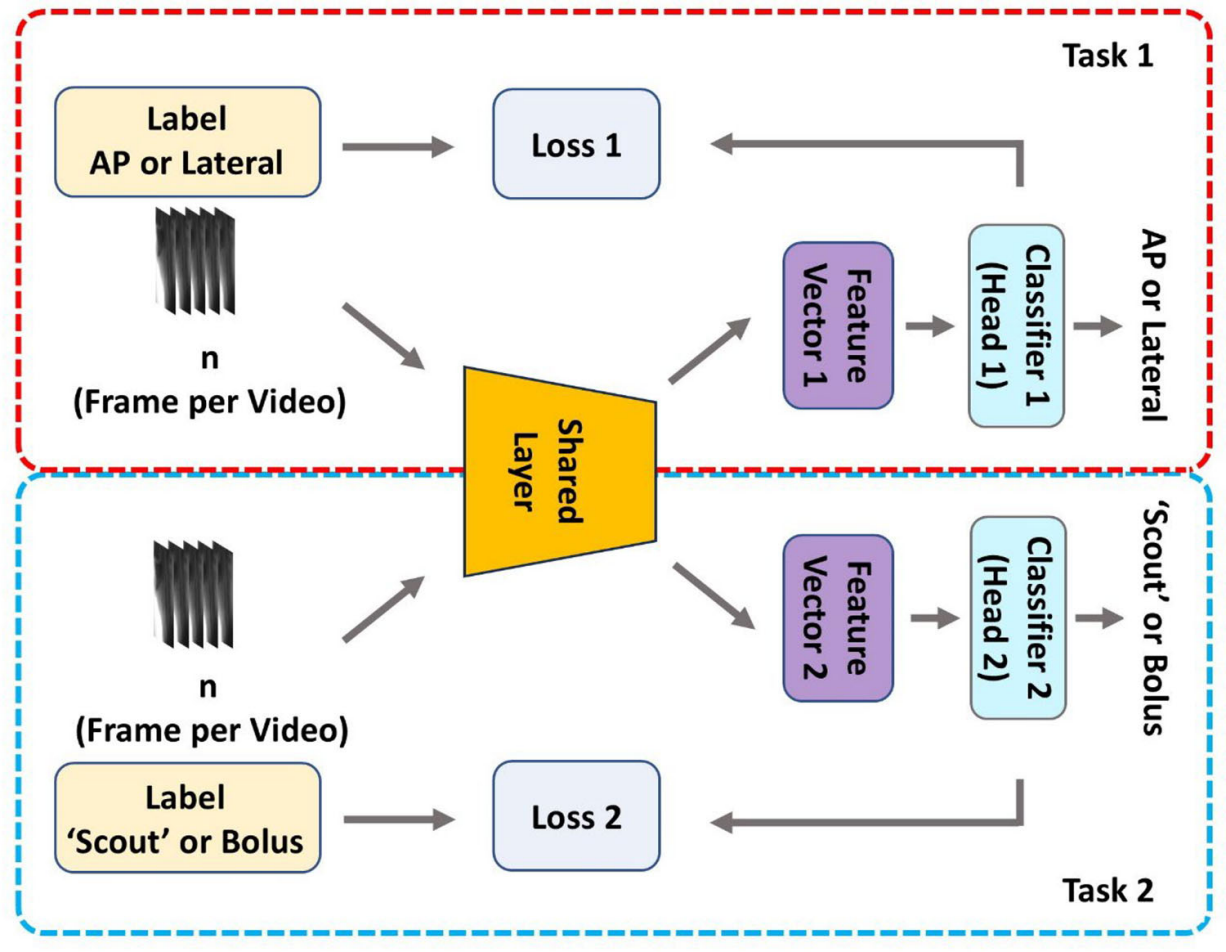
Multi-task learning framework for automatic classification of MBS video orientation and scout video identification

Our deep learning framework adopted a shared CNN based on ResNet50 for frame-level feature extraction. Specifically, we removed the original classification head (a fully connected layer with 2048 inputs and 1000 outputs) of ResNet50 to retain only the convolutional layers, which served as the shared feature extractor across tasks. This component produced a 2048-dimensional feature vector for each frame and corresponds to the "CNN feature extractor" illustrated in Fig. 2. The extracted features were then passed into two independent task-specific fully connected (FC) layers—referred to as task-specific classifiers/heads—to perform classification for each respective task, as depicted in Fig. 3.

To train the model, we employed an alternating-task strategy rather than minimizing the sum of the two losses. Specifically, each training epoch is dedicated to a single task. During that epoch, only the corresponding task-specific head and the shared CNN were updated using the cross-entropy loss. In the subsequent epoch, we switch to the other task, updating the shared CNN and the other head accordingly. This strategy avoids issues related to loss balancing between tasks. Additionally, this strategy can address the training data issue, because the two tasks involved, although related, different training datasets.

A key advantage of this approach is its flexibility in handling mixed input pipelines: It can support frame-wise data for one task and video-wise data for the other, making it adaptable to various clinical scenarios. We also maintained a consistent number of frames per video (parameter *n*) across both classification tasks.

### Other setups

All MBS video frames were in grayscale and converted to three-channel images for pretrained models. To enhance model robustness, data augmentation was applied during training, including random scaling and cropping (scale range 0.8–1.0, aspect ratio 0.6–1.667). All input images were then resized to [512, 512]. We used random horizontal flips but skipped vertical flips to preserve the upright patient orientation typical in MBS exams. For video-wise analysis, the same augmentation was consistently applied to all frames in a single video. No augmentations were used for the validation or testing sets. We employed accuracy (ACC), sensitivity (SEN), specificity (SPE), F1 score, and Area under the ROC Curve (AUC) as the primary metrics to assess model performance on the testing set. Training employed a Stochastic Gradient Descent (SGD) optimizer for each task’s cross-entropy loss (learning rate = 0.0001, momentum = 0.9). The batch size was 16, corresponding to the number of videos. To address the data imbalance issue, the Synthetic Minority Over-sampling Technique (SMOTE) was applied to the training data. This study was conducted on a Kubernetes GPU cluster, where each node had 128 physical CPUs, 1 TB of memory, and 8 Nvidia A100 GPUs**.**

## Results

### Performance of single task learning

**Fig. 4:**
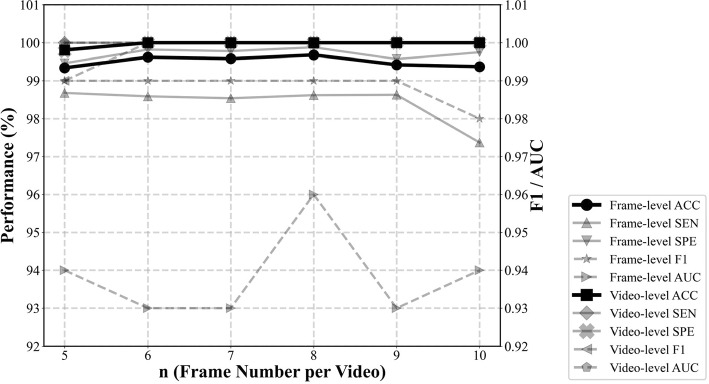
Performance metrics of the video orientation classification

**Fig. 5:**
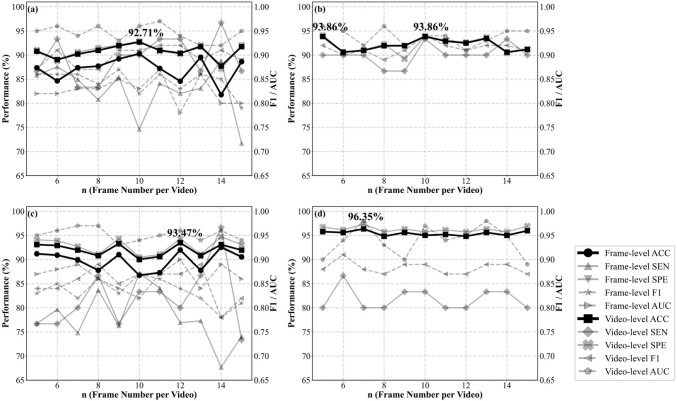
Performance metrics of ‘scout’/bolus swallowing classification. (**a**) single-task learning, with frames randomly selected from each video; (**b**) single-task learning with video-wise analysis; (**c**) multi-task learning, with frames randomly selected from each video; and (**d**) multi-task learning with video-wise analysis

In the experimental stage, we first trained single-task models separately, with no shared components. For both tasks, we used a frame-wise analysis, treating each frame independently for video orientation and ‘scout’/bolus classification. The results of orientation classification are shown in Fig. 4. Frame-level accuracy began at 99.34% for *n* = 5, fluctuated slightly, and peaked at 99.68% for *n* = 8; F1 scores remained around 0.99. Notably, at the video level, accuracy reached 100% and an F1 score of 1.00 from *n* = 6 to 10, indicating video-level performance was not sensitive to the frame number for this task. Figure 5a shows single-task learning results for ‘scout’/bolus classification using frame-wise analysis. Frame-level accuracy started at 87.37% for *n* = 5, fluctuated across n, and reached 90.26% at *n* = 10. F1 scores followed a similar trend, beginning at 0.86 (*n* = 5), peaking at 0.87 (*n* = 9), then dipping to 0.79 at *n* = 15. At the video level, the model achieved its highest video-level accuracy of 92.71% and F1 score of 0.91 at *n* = 10.

Next, we performed single-task learning using video-wise analysis for the ‘scout’/bolus classification task only, because of the strong frame-wise results shown in Fig. 4 for AP-lateral classification. As shown in Fig. 5b, accuracy peaked at 93.86% for *n* = 5 and 10. F1 scores followed a similar pattern, starting at 0.92 (*n* = 5) and reaching 0.94 at *n* = 10. Comparing Fig. 5a and b, the video-wise approach outperformed the frame-wise approach, achieving about 1% higher accuracy at its peak (*n* = 10).

### Performance of multi-task learning

In this section, we applied multi-task learning to video orientation classification and ‘scout’/bolus classification, and we only focused on the second task, as shown in Fig. 5c and d. Under the frame-wise approach (Fig. 5c), frame-level accuracy ranged from 86.74 to 92.54%, with corresponding F1 scores showing similar variability. Video-level metrics were generally higher, peaking at 93.47% accuracy (*n* = 12) and 0.89 F1 (*n* = 8, 13). Compared to single-task learning with the same frame-wise pipeline (Fig. 5a), multi-task learning provided an overall enhancement in video-level accuracy, increasing from a peak of 92.71% (*n* = 10) to 93.47% (*n* = 12). Figure 5d presents the performance metrics for ‘scout’/bolus swallowing classification using a multi-task learning framework, where we applied the video-level analysis as the data pipeline. The results demonstrated high video-level accuracy and F1 scores across varying frame numbers per video (*n*). The video-wise analysis achieved improved accuracy compared with the frame-wise analysis, and the highest accuracy observed is 96.35% at *n* = 7. Moreover, the multi-task learning consistently achieved higher video-level accuracy than single-task learning, as shown in Fig. 5b. The F1 score reached the highest value of 0.91, occurring at *n* = 6 frames per video, which is slightly lower than the highest F1 score in Fig. 5b.

### Statistical analysis

To further evaluate the performance differences between the setups, we conducted paired *t*-tests on the video-level accuracy results for the ‘scout’ vs. bolus classification task across all methods and calculated the mean values. As shown in Table 3, multi-task learning with video-wise analysis achieved the highest mean accuracy (95.43%) and statistically outperformed all other approaches (*p* < 0.001 in all comparisons). An alpha level of 0.05 was used to determine statistical significance.

**Table 3:** Paired t-test results comparing video-level accuracies of the ‘scout’ vs. bolus classification task across four setups

*p* value	Single-task learning with frame-wise analysis (Mean value 90.75%)	Single-task learning with video-wise analysis (Mean value 92.17%)	Multi-task learning, with frame-wise analysis (Mean value 89.78%)
Single-task learning with video-wise analysis (Mean value 92.17%)	0.007	–	–
Multi-task learning, with frame-wise analysis (Mean value 89.78%)	0.413	0.019	–
Multi-task learning with video-wise analysis (Mean value 95.43%)	< 0.001	< 0.001	< 0.001

Single-task learning with video-wise analysis (mean = 92.17%) also significantly outperformed single-task learning with frame-wise analysis (mean = 90.75%, *p* = 0.007) and multi-task learning with frame-wise analysis (mean = 89.78%, *p* = 0.019). Additionally, the comparison between single-task and multi-task learning under the frame-wise analysis condition did not yield a statistically significant difference (*p* = 0.413), indicating similar performance between the two in the frame-wise setup.

## Discussion

In MBS studies, effectively identifying AP and lateral orientations in video series and filtering out scout videos constitute foundational tasks for efficient clinical interpretation. These tasks not only facilitate precise swallowing evaluation for patients but might also significantly enhance the workflow efficiency for healthcare professionals. This study introduces a computational approach designed to automate this critical video pre-sorting process, thereby optimizing the MBS analysis workflow. Our results demonstrate the possibility of deploying deep learning approaches for automating these processes, showcasing remarkably high accuracy rates for both tasks. This study, therefore, highlights the potential of deep learning in streamlining the very first step of MBS study analysis, paving the way for enhanced automation in the evaluation of swallowing disorders.

In our analysis, the classification of AP-lateral orientation through frame-wise analysis achieved a remarkable 100% accuracy at the video level, indicating that identifying the AP-lateral direction is not a formidable challenge for deep learning. For this task, the extracted features are salient and readily identifiable, enabling robust orientation recognition. The consistency of this high accuracy across a range of frame numbers (*n*) from 5 to 10 suggests minimal sensitivity to changes in the number of frames per video clip. Therefore, it is reasonable to assert that the method would remain unaffected by an increase in the number of frames per video, such as 11–15, which were tested in the ‘scout’ video identification task. This method holds promise for facilitating frame-by-frame analysis in scenarios where AP-lateral MBS frames are captured within a single continuous video sequence. Given that frame-wise analysis has achieved over 99% accuracy (Fig. 4), our approach could separate the imaging segment according to the orientation and significantly assist the MBS study evaluations.

Contrary to the high performance achieved in the AP-lateral classification task, the task of identifying scout videos using frame-wise analysis within a single-task learning framework yielded lower accuracy levels (peak: 92.71%, mean: 90.75%). This variation suggests that the features critical for distinguishing scout videos are less salient at the frame level compared to those required for AP-lateral orientation differentiation. Additionally, our analysis revealed that performance metrics varied with changes in the number of frames per video, indicating a sensitivity to the batch size used during training. Given that our training setup configured the batch size to correspond with the number of videos, the total number of frames (images) processed by the model in each iteration was the product of batch size and the number of frames (*n*). Despite not achieving the best performance, the results from this setup preliminarily demonstrate deep learning’s capacity to segregate non-diagnostic scout videos from diagnostic content in MBS studies.

The task of scout video identification within a single-task learning framework revealed notable differences in performance between video-wise and frame-wise analyses. Specifically, video-wise analysis (Fig. 5b) demonstrated more than 1% higher accuracy (peak: 93.86%, mean: 92.17%) in video-level performance compared to frame-wise analysis (peak: 92.71%, mean: 90.75%, Fig. 5 a) with *p* = 0.007. This distinction underscores the importance of considering the inter-frame relationships within a video clip (segment) for accurately identifying non-diagnostic swallowing videos, a consideration that diverges from the approach required for orientation classification tasks. The underlying reason for this disparity lies in the fundamental difference between scout and bolus swallowing videos: the presence or absence of the food bolus (contrast agent) throughout all the video frames. Unlike scout videos, which consistently lack bolus presence across all frames, bolus swallowing videos predominantly feature the bolus throughout the video frames. Often, bolus swallows begin recording at the oral stage [23] (frame displaying the bolus within the oral cavity). However, there are instances where the recording starts before the bolus is visible to the X-ray camera, resulting in some initial frames without the bolus, similar to the frames found in scout videos. This scenario presents a challenge for frame-wise analysis, leading to suboptimal results, and multi-task and single-task learning showed comparable performance under the frame-wise analysis setting (*p* = 0.413). The advantage of video-wise analysis lies in its ability to incorporate the temporal sequence of frames, enhancing the model’s capability to differentiate videos based on the overall pattern of bolus presence. By accounting for the context provided by the frames sampled at fixed interval, video-wise analysis improves accuracy. This framework effectively captures the dynamic nature of swallowing processes, thereby enabling more precise classification of MBS video content.

In the context of ‘scout’ video identification utilizing frame-wise analysis, the adoption of a multi-task learning with video-wise framework yielded an improvement in accuracy over single-task learning approaches (*p* < 0.001). This enhancement suggests that multi-task learning analysis is capable of extracting more relevant features from MBS frames than its single-task counterpart, thereby offering a more refined analysis. Despite this advancement, frame-wise analysis within a multi-task learning paradigm did not surpass the performance of single-task learning when employing a video-wise analysis approach (peak: 93.47%, mean: 89.78% vs. peak: 93.86%, mean: 92.17%: *p* = 0.019). This outcome underscores the significance of the inter-frame relationships over the analysis of individual frames in the identification of scout videos. The comparative analysis indicates that understanding the temporal continuity and context between frames is crucial for accurately classifying scout videos, highlighting the importance of video-wise analysis in capturing the dynamic aspects of MBS studies.

In our exploration of ‘scout’ video identification utilizing video-wise analysis, the implementation of multi-task learning has markedly enhanced accuracy beyond the results observed in previous setups. Notably, this setup achieved the highest mean accuracy (95.43%) and significantly outperformed all other configurations (*p* < 0.001). This signifies the potential of multi-task learning to elevate the performance of scout identification by concurrently leveraging the orientation classification task. The multi-task learning approach facilitates the CNN model in extracting more robust features, emphasizing the importance of capturing the inter-frame connectivity within individual videos to augment overall accuracy. While the F1 scores obtained through multi-task learning did not uniformly exceed those achieved via single-task learning, the emphasis on video-level accuracy remains crucial. In clinical practice, the precise identification of each video is of equal importance. The consistently elevated accuracy levels attained with multi-task learning underscore its enhanced efficacy and underscore its promise for transforming the analysis of ‘scout’/bolus swallowing videos within clinical frameworks. This advancement in multi-task learning not only demonstrates its capability to improve diagnostic accuracy but also highlights its potential to streamline and refine the evaluation process in clinical settings, ensuring a more efficient assessment of swallowing disorders. While the proposed model achieved human-level accuracy in the AP-lateral classification task, it did not yet reach the same level of accuracy as human raters for the ‘scout’–bolus classification task (> 99%). This represents a limitation of the current study and suggests that more sophisticated deep learning architectures may be required to further improve performance in this area.

This work also casts new light on advancing deep learning applications in MBS studies. With a growing trend toward utilizing deep learning for automating clinical outcomes in diagnostic MBS videos, current endeavors mainly concentrate on detecting anatomical landmarks used to measure elements of swallowing physiology and segmenting the bolus [24]. These efforts typically analyze individual frames, similar to our study’s frame-wise analysis, but skipped the temporal coherence. Our findings suggest the possibility of unifying these diverse efforts under a singular CNN model through a multi-task learning approach. This unified strategy offers two key advantages: It enhances the model’s capacity to interpret semantic information within MBS images and identify more robust features, and it provides a solution to the challenge of acquiring extensive, reliable clinical annotations. By integrating annotations across multiple tasks, this approach can expand the training dataset, thereby bolstering the model’s generalization capabilities. This method not only simplifies the analytical framework but also holds the promise of elevating the accuracy and comprehensiveness of MBS study interpretations, offering substantial promise to clinical diagnostics and patient care. Additionally, the challenges and insights from applying deep learning to MBS scout images are relevant across other medical imaging, where ‘scout’ images are common. This underscores the need for adaptable deep learning strategies that address pre-sorting in various imaging studies, promoting broader advancements in automated medical diagnostics.

## Conclusion

This study showcases a deep learning strategy for automatically distinguishing patient orientation and identifying ‘scout’ videos within MBS studies, aiming to enhance the workflow efficiency in the interpretation of radiographic swallowing function assessments. By accurately determining AP-lateral orientations and employing a multi-task learning approach, our methods improve the identification of ‘scout’ videos, demonstrating potential for automating the initial image sorting step in the analysis phase of MBS studies. The effective use of video-wise analysis to incorporate inter-frame information highlights the critical role of analyzing the dynamic nature of swallowing processes. Implementing this technology has the potential to simplify MBS study review and labeling, thereby supporting more efficient clinical workflows. Additionally, our model lays the groundwork for achieving fully automated MBS analysis, supporting the advancement of deep learning-driven studies in this domain. Our results demonstrate the feasibility of applying deep learning to optimize pre-analysis steps in radiographic swallowing studies, offering a promising direction for further diagnostic applications.

## Data Availability

In accordance with the Final NIH Policy for Data Management and Sharing, NOT-OD-21–013, anonymized tabular analytic data that support the findings of this study are openly available for review purpose in an NIH-supported generalist scientific data repository (figshare) at https://figshare.com/s/f9e8f5b84b6b9b9c08fa no later than the time of an associated peer-reviewed publication; while public data are embargoed pending peer review, the data are available upon request pre-peer review through email to the corresponding author.
